# A novel recombinant chimeric bio-adhesive protein consisting of mussel foot protein 3, 5, gas vesicle protein A, and CsgA curli protein expressed in *Pichia pastoris*

**DOI:** 10.1186/s13568-022-01362-5

**Published:** 2022-02-27

**Authors:** Nazanin Bolghari, Hosein Shahsavarani, Masoumeh Anvari, Hadi Habibollahi

**Affiliations:** 1 Department of Biology, Islamic Azad University, Rasht branch, Rasht, Iran; 2 Department of Cell and Molecular Sciences, Faculty of Life Sciences and Biotechnology, Shahid Beheshti University, Tehran, Iran

**Keywords:** Bio-adhesive proteins, Fusion proteins, GvpA, CsgA, Mfps, Underwater adhesives

## Abstract

Despite various efforts to produce potent recombinant bio-adhesive proteins for medical purposes, efficient production of a safe and feasible bio-glue is not yet a commercial reality due to the weak properties or low expression levels. Here, a feasible expression system has been developed to produce strong recombinant fusion bioinspired protein using mussel foot protein 3 and 5 (Mfps) along with gas vesicle protein A (GvpA) of *Anabaena flos-aquae,* and a curli protein CsgA from *E. coli*, expressed under the control of alcohol oxidase (AOX1) promoter for high-level production in yeast *P. pastoris* using pPICZα vector. Purified chimeric proteins were first evaluated using western blotting, and their remaining dihydroxyphenylalanine (DOPA) was measured in the modified proteins by NBT assay. We further elucidated the mechanistic properties of obtained adhesive protein assembly in various pH levels based on its different subunits using atomic force microscopy (AFM) when adsorbed onto the mica surface. We found that both combinational structural features of subunits and post-translational changes during expression in yeast host have led to potent adherence due to higher DOPA residues specially in acidic condition and tetrad complex which is higher than that of earlier reports in prokaryotic systems. We believe that our obtained chimeric protein resulted from the fusion of GvpA and CsgA proteins with DOPA-containing Mfp proteins, expressed in the methylotrophic yeast, *P. pastoris*, not only presents a candidate for future biomedical applications but also provides novel biological clues used for high-performance bioinspired biomaterial designation.

**Graphical Abstract:**

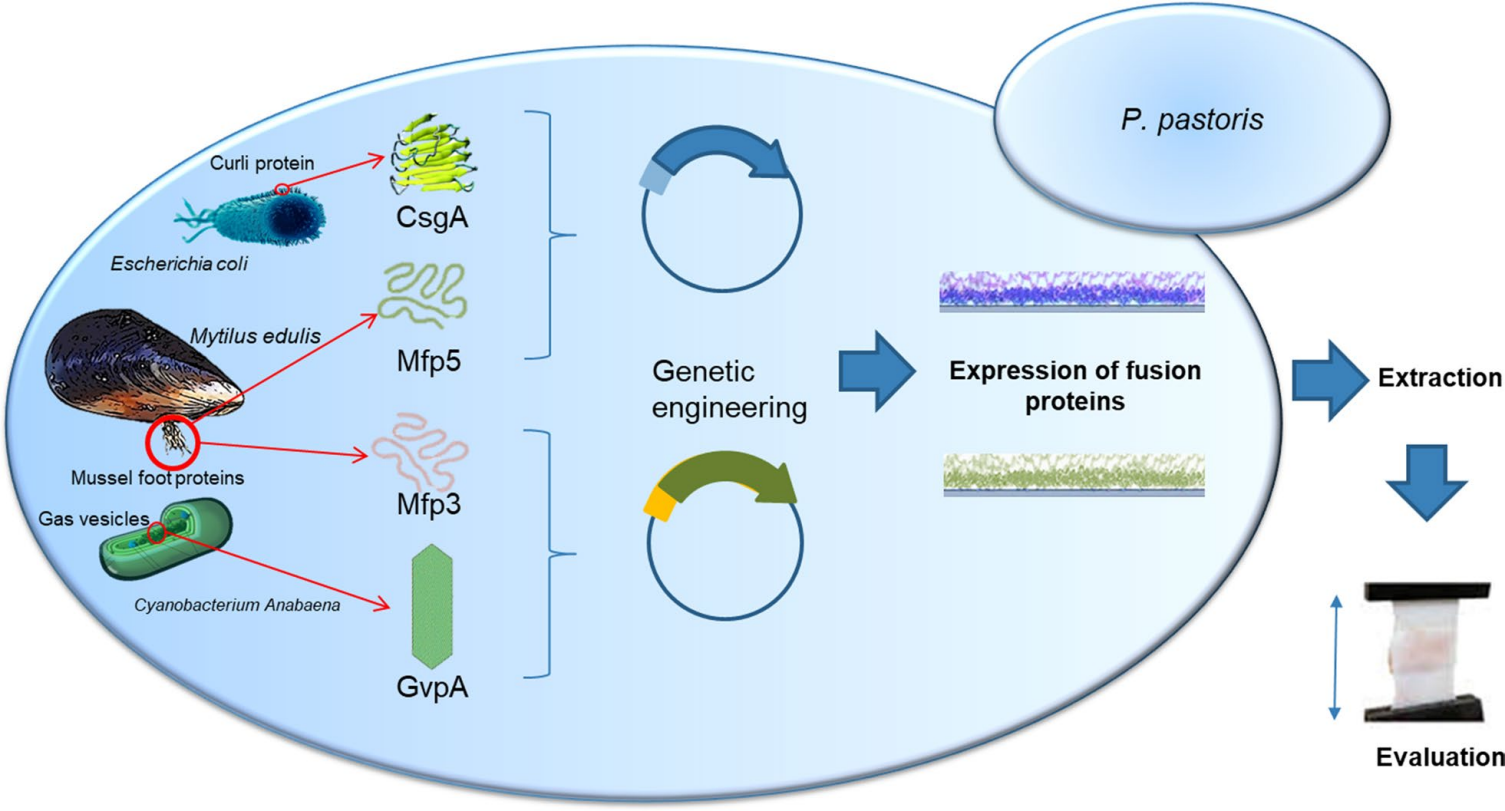

## Keypoints

- A chimeric bioinspired adhesive protein has been generated for use in wet condition
- Enhanced adhesion strength of chimeric protein due to the curli proteins GvpA and CsgA
- Amyloid structure of GvpA and CsgA led to efficient self-assembly of obtained protein

## Introduction

The use of adhesive material for the repairment of tissues following surgery or trauma has attracted much attention and is likely to replace traditional methods such as suture, wiring or staples. Most of the surgical adhesives used in operations are cyanoacrylate derivatives, which still have numerous limitations like cytotoxicity, inflammatory reaction or operational complexities (Choi et al. 2011). Another type of wound closure generally applied in surgery is animal-derived glues, which have numerous issues such as, safety concerns or non-adequate bond toughness. In spite of tremendous efforts to develop adhesives with broad applications in different tissues or non-tissue surfaces, the safe and outstanding material for tissue adhesion could not yet reach an industrial reality to meet different clinical requirements (Rebnegger et al. 2014).

Therefore, bioinspired recombinant bio-adhesive recombinant protein production has been suggested to address the aforementioned challenges with less drawbacks such as safety issues, need for further cosmetic operation or any allergic reactions (Cha et al. 2009). Recent advances in biomimetic research have recently led to the design of various novel recombinant proteinaceous glues capable of satisfying specific demands of both biomedical and technical fields through cloning and expression of the natural adhesive proteins (Deming 1999). Numerous marine organisms such as mussels and barnacles are great sources of novel water-resistant adhesive biomaterials that are expected to make adequate bonding toughness that support physiological conditions, where tissues are exposed to excessive moisture and body fluids due to their waterproof properties (Zhao and Waite 2006; Choi et al. 2011). These waterborne bio-adhesives were secreted to form holdfast filaments known as the byssus, where they are specifically adapted for functions such as wet adhesion and protective covers (Deming 1999; Priemel et al., 2017).

Mussel foot proteins (Mfp2, -3S, -3F, -4, -5 and -6) are a group of bioinspired adhesive proteins in the byssal adhesive plaque of the *Mytilus* species that was produced, secreted and solidified to provide tenacious anchoring to different substrates in harsh underwater environment (﻿Rzepecki et al. 1992; Yu et al., 2013). Amongst them, Mfp3 and Mfp5 play a significant role in adhesion process. Mfp3 is a protein with low molecular weight in two fast and slow versions, with tyrosine residues that are post-translationally modified to DOPA whereas, Mfp5 is small, with high glycine and lysine content (Yu et al. 2011; Rahimnejad and Zhong 2017; Rebnegger et al. 2014). In spite of extensive research on characterization and recombinant production of Mfp3 and Mfp5, a functional, cost-effective and self-assembled glue has not yet been achieved. Imitation of the unique wet adhesion ability of proteinaceous adhesives with stronger intermolecular interactions in surface adhesion properties has been achieved via the combination of prokaryotic curli proteins such as CsgA from *E. coli* and Mfp proteins. However, the pathogenicity of its source still limited its application for biomedical purposes.

It thus suggested that using another safe cross-linking protein from a nonpathogenic origin will possibly improve its adhesion for biomedical applications without any safety concerns. GvpA is the amyloid-like fiber in *Dolichospermum flosaquae* that sustains the hydrostatic pressure experienced by the cells in the ocean (Pfeifer 2012; Hayes et al. 1992). Exploiting GvpA can play the cohesive role to maintain the structure of obtaining chimeric protein to the acceptable stability/toughness of the adhesion level for use in wet or humid environments such as the human body. We have recently reported a recombinant chimeric bio-adhesive protein by fusion of mussel foot proteins and GvpA in *E. coli* (Iranpour et al. 2021). Although prokaryotic expression systems are impressive and convenient with straightforward protocols, these cell factories have some limitations for the production of biomolecules in industrial scale. Thus, exploiting yeasts especially *P. pastoris* has been suggested to overcome the difficulties associated with bacterial expression systems such as intracellular aggregation and misfolding, lack of posttranslational modification, or degradation of heterologous proteins by proteases (Baumann et al. 2011). Moreover, it is also considered a unique host for recombinant protein production mainly because of its high growth rate, ease of its genetic material manipulation, secretory expression, and proper glycosylation of recombinant target proteins in the precise sites which is necessary for protein stability (Mattanovich et al. 2009). In the present study, a genetic engineering approach has been used to express a novel combinational bio-adhesive fusion protein including mussel foot proteins 3 and 5 (Mfp3, Mfp5) of *Mytilus Californianus* and gas vesicle protein A (GvpA) of *Dolichospermum flosaquae* and CsgA curli protein of *E. coli* in *P. pastoris*. It is expected that self-assembly of GvpA and CsgA as amyloidogenic proteins, can improve adhesion of their chimeric combination with Mfp3 and Mfp5 to make strong amyloid nanofibers with DOPA residue on the outside of amyloid scaffold. Moreover, some degree of process optimization such as pH level adjustment has been performed and post-translational features of obtained fused recombinant proteins were examined. Finally, we have assessed and compared adhesion properties of the fused adhesive proteins separately.

## Materials and methods

### Strains, plasmids, enzymes, reagents

*Escherichia coli* strain TOP 10 (Invitrogen) was used as a host for molecular cloning of DNA in pPICZα (Invitrogen) and propagation of recombinant expression vectors. *P. pastoris* strain GS115 (Invitrogen) was used for heterologous protein expression. All media and protocols for *P. pastoris* are described in the *P. pastoris* expression manual (Invitrogen). All of molecular biology enzymes, antibiotics, DNase/RNase-free, and distilled water were purchased from Thermo Fisher Scientific Corporation, USA. Other chemicals were procured from Sigma-Aldrich Corporation, USA.

### DNA preparation and cloning of Mfp5-CsgA and Mfp3-GvpA

The genes complete nucleotide sequences were retrieved from the NCBI database (Table 1). The coding sequences of the Mfp5-CsgA and Mfp3-GvpA genes including a GS linker were inserted between *Eco*RI and *XhoI* restriction sites fused to a histidine-tag in the pPICZα vector and synthesized (Biomatik Co, Canada). To ensure correct insertion of Mfp5-CsgA and Mfp3-GvpA, polymerase chain reaction (PCR) was performed using primers listed in Table 2. The recombinant plasmid was transformed into competent *E. coli* TOP10F′ by using heat shock method. (Dolgin 2013). It was grown in either Luria–Bertani broth or on Luria–Bertani agar, supplemented with zeocin (50 μg/mL) when required. Several positive clones were selected and sent to a commercial laboratory for sequencing to confirm the complete nucleotide sequence of the gene insert. Transformation of *P. pastoris* with the recombinant pPICZα-Mfp was adopted according to the manufacturer’s protocol of the EasySelect™ *P. pastoris* Expression Kit (Invitrogen, Carlsbad, CA) (Safder et al. 2018). Positive recombinant *P. pastoris* clones were selected for expression.

**Table 1:** List of proteins utilized in this study, their species' origin, and accession numbers

Name of the protein	Source	Accession No. of proteins
Mussel foot protein 3 (Mfp3)	*Mytilus californianus*	GenBank AAY29126.1
Mussel foot protein 5 (Mfp5)	*Mytilus californianus*	GenBank ABE01084.1
Gas vesicle protein A (GvpA)	*Dolichospermum flosaquae*	GenBank AAA82497.1
CsgA	*Escherichia coli*	GenBank ACB15778.1

**Table 2:** Oligonucleotide sequences designed as the and reverse primers for the Mfp5-CsgA and Mfp3- GvpA genes

Primer	Sequence
Forward Mfp5-CsgA	3′GACTGGTTCCAATTGACAAGC5′
Reverse Mfp5-CsgA	5′GCAAATGGCATTCTGACATCC3′
Forward Mfp3-GvpA	3′GACTGGTTCCAATTGACAAGC5′
Reverse Mfp3-GvpA	5′GCAAATGGCATTCTGACATCC3 ′

### Transform genes to *Pichia pastoris competent cells*

The selected expression plasmid was linearized with *Sac* I (New England BioLabs, USA) and then transformed into *P. pastoris* by electroporation. Transformants were first screened from YPDS (1% yeast extract, 2% peptone, 2% dextrose, 1 M sorbitol, 2% agar) plates containing Zeocin™ at a final concentration of 100 μg mL^−1^, then on YPDZ plates (1% yeast extract, 1% peptone, 1% dextrose and 2% agar containing Zeocin at final concentrations of 150, 300, and 500 μg mL^−1^) in order to screen for higher copy numbers of the targeted gene. Recombinant strains producing mannan endo-1, 4-β-mannosidase were further confirmed by BMGY-Azo plates (1% yeast extract, 2% peptone, 100 mM potassium phosphate pH 6.0, 1.34% YNB, 4 × 10^–5^% biotin, 0.5% methanol, 2% agar and 0.3% Azo-carob galactomannan).

### Expression in *P. pastoris*

YPD growth medium containing yeast extract (1%), peptone (2%), and glucose (2%) was inoculated with single colonies of *P. pastoris* (200 ml). The cells were cultured at 30 °C for 36 h. The cells were then collected by centrifugation for 10 min at 3000 ×*g*, 4 °C and resuspended to an approximate OD_600_ of 2 in 300 ml of BMGY Buffered glycerol–complex medium (0.1% yeast extract, 0.2% peptone, 100 mM potassium phosphate Ph 6.0, 1.34% YNB, 4 × 10 biotin 1% glycerol or 0.5% methanol) were supplemented with 0.5 μM δ-aminolevulinic acid, trace element (250 μl/liter) and zeocin (100 ng/ml). Cells were grown at 25–26 °C at agitation rate of 200 rpm and induced for 96 h by adding methanol (0.5%) every 24 h. After 96 h, the cells were harvested by centrifugation at 3000 ×*g* for 10 min, at 4 °C Cells were resuspended in breaking buffer (50 mM sodium phosphate, pH 7.4, 1 mM EDTA, 5% (v/v) glycerol, 2 mM DTT, and 1 mM protease inhibitor). The cell suspension mixture was assorted with an equal volume of acid-washed glass beads (0.5 ~ 0.75 mm in diameter) and disrupted by vortexing (830 s at 4 °C with cooling on ice for 30 s between the cycles). The lysate was separated from cell debris and glass beads by centrifugation at 10,000 ×*g* for 8 min at 4℃. The supernatant was centrifuged at 20,000 ×*g* at 4 °Cfor 1 h and then, the microsomal pellet was resuspended in breaking buffer and stored at − 80 °C.

### SDS – PAGE and Western blot

Extracted proteins were separated by sodium dodecyl sulfate–polyacrylamide gel electrophoresis (SDS-PAGE) on 10% polyacrylamide gels. Electrophoresis was performed at 40 mA and 100 V and after that, SDS-PAGE gel was stained with Coomassie Brilliant Blue solution (Bio-Rad). For Western blot, the gel was electrophoretically transferred onto polyvinylidene difluoride (PVDF) membrane. Membranes were blocked in 5% (w/v) non-fat dried milk in Tris-buffered saline with 0.1% tween 20 (TBST) for 1 h at 4 ℃. Membranes were then incubated overnight with anti-his-tag antibody at a 1:1000 dilution in TBST. The membrane was incubated with horseradish peroxidase (HRP)-conjugated goat anti-mouse IgG antibody. Proteins were visualized by an enhanced chemiluminescence method using ChemiDoc XRS (The Multi-Copy Pichia Expression Kit, 2010).

### Purification of proteins

For protein purification, we used the affinity HisTag purification method. For the purification of His-tag fused AAT, the supernatant was applied to a nickel-immobilized chelating sepharose fast flow column (Amersham, Biosciences). For this purpose, supernatant was first diluted with an equal volume of 2× binding buffer (50 mM NaH_2_PO_4_, 500 mM NaCl, 10 mM imidazole, pH = 7.4) and then loaded on to the column. After passing the wash buffer (50 mM NaH_2_PO_4_, 500 mM NaCl, a gradient of imidazole from 20 to 40 mM, and 0.05% (*v/v*) Tween 20, pH = 7.4) through the column, the resin-bounded recombinant AAT was eluted with elution buffer (50 mM NaH_2_PO_4_, 500 mM NaCl, 250 mM imidazole, and 0.05% (*v/v*) Tween 20, pH = 7.4).

### NBT and ABDS

DOPA, a hydroxylated form of tyrosine, has a key role for adhesion in underwater conditions because of its reversible adhesive properties and strong non-covalent bond with half-strong covalent bonding with a wet metal oxide surface (Rahimnejad and Zhong 2017). The conversion of tyrosine to DOPA during post-translational modification is important, since it is responsible for directly adhering proteins to a different surface. Because DOPA and dopaquinone can be detected by redox cling staining using glycine and NBT, the NBT staining method is widely used for detecting DOPA in MAPs (Zhang et al. 2017a, b). Unmodified *E. coli* derived Mfp5- CsgA and Mfp3-GvpA (Mfp5- CsgA and Mfp3-GvpA before expiration in *Pichia pastoris*) and in vitro tyrosinase modified *Pichia pastoris* derived Mfp5- CsgA and Mfp3-GvpA were used as negative and positive controls, respectively. Moreover, Acid-borate difference spectrum analysis was performed to assess the amount of DOPA in modified bio-adhesive proteins. For this purpose, while the absorbance of DOPA in acid conditions is 280 nm, alteration of the wavelengths due to the formation of diol-borate at pH = 7–12 (287 nm) was monitored.

### Mass spectrometry

Matrix-assisted laser desorption/ionization time-of-flight (MALDI-TOF) mass spectrometry was performed using an ultra-flex treme mass spectrometer (Bruker, Leiderdorp, The Netherlands). Proteins were desalted using Micro Bio-Spin P-6 columns (Bio-Rad, Veenendaal, The Netherlands), and samples were prepared by the dried droplet method on a 600 µm Anchor Chip target (Bruker, Leiderdorp, The Netherlands), using 8 mg mL^−1^ 2,5-dihydroxyacetophenone, 1.5 mg mL^−1^ diammonium hydrogen citrate, 25% (v/v) ethanol and 3% (v/v) trifluoroacetic acid as matrix. Spectra was derived from ten 500-shot (1000 Hz) acquisitions taken at non-overlapping locations across the sample. Wide mass-range measurements were made in the positive linear mode, with ion source 1, 25.0 kV; ion source 2, 23.3 kV; lens, 6.5 kV; pulsed ion extraction, 680 ns. Detailed analyses of glycoproteins in the ~ 31–51 kDa range were done with ion source 1, 20.0 kV; ion source 2, 18.4 kV; lens, 6.2 kV; pulsed ion extraction, 450 ns, and spectra were derived from ten 1000-shot (1000 Hz) acquisitions. Protein Calibration Standard II (Bruker, Leiderdorp, The Netherlands) was used for external calibration.

### Surface analysis

Surface topography and mean average surface roughness (Ra) were examined by atomic force microscopy (AFM) (DME*Dualscope* c-26, Denmark). To perform the surface topography test, we tested samples Mfp3-GvpA and Mfp5-CsgA in both pH 2.6 and pH 5.5**.** For this purpose, 20 µl of the purified samples of Mfp3-GvpA and Mfp5-CsgA was poured onto the mica surface with 1 M acetic acid and placed in an AFM device after drying. In two pH 2.6 and 5.5 (Huang et al. 2012a, b; Urushida et al. 2007a, b).

## Results

### Genomic Integration of the pPICZα vector

**Fig. 1:**
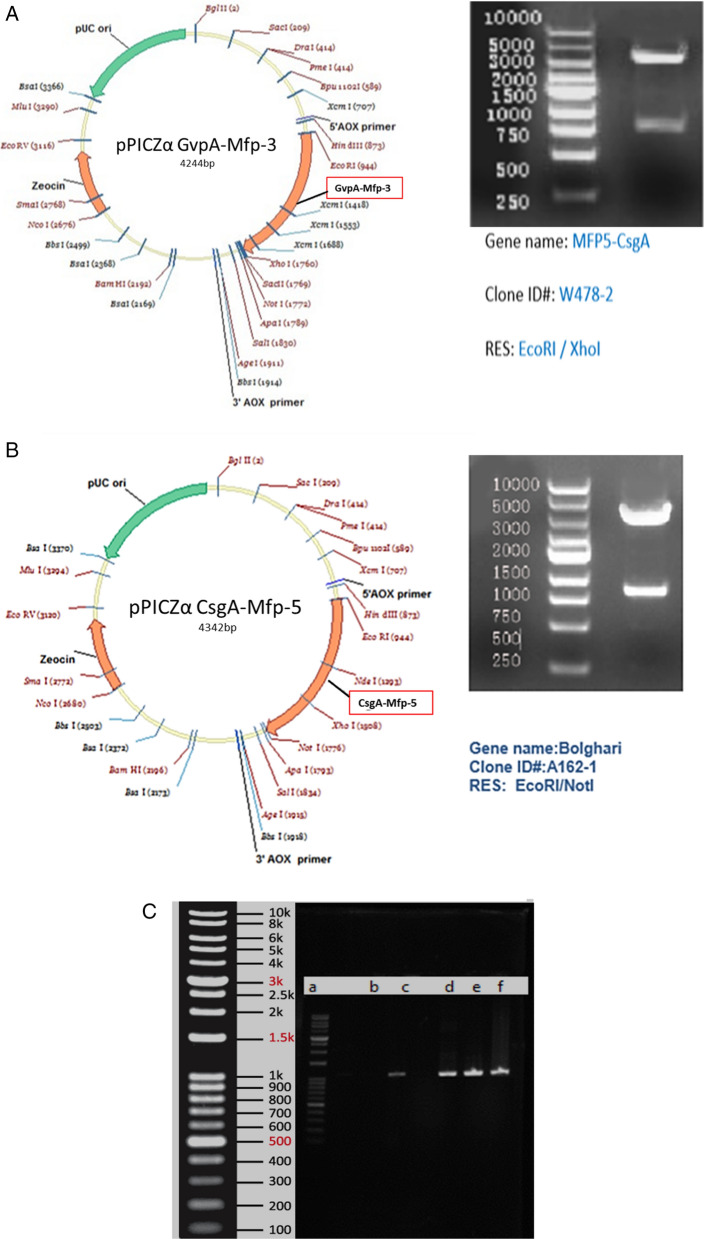
Map of the pPICZα A-GvpA-Mfp3 (**A**) and pPICZα A-CsgA-Mfp5 plasmid construction (**B**). Gene sequences were inserted between *Eco*RI and *Xho*I, *Eco*RI and *Not*I restriction sites, respectively. Proof of the successful transfection and presence of the desired chimeric gene in yeast by PCR amplification (**C**): Mfp5- CsgA (Lane c), GvpA-Mfp3 (Lanes d, e, f), and DNA marker (Lane a)

MPF5-CsgA and MPF3-GvpA genes were cloned into the yeast cell of *Pichia pastoris* and produced the desired proteins. After enzymatic digestion, two 750 bp bands (Gene) and 2000 bp (pPICZα vector) were observed (Fig. 1).

### Expression of recombinant chimeric proteins and SDS-PAGE analysis

**Fig. 2:**
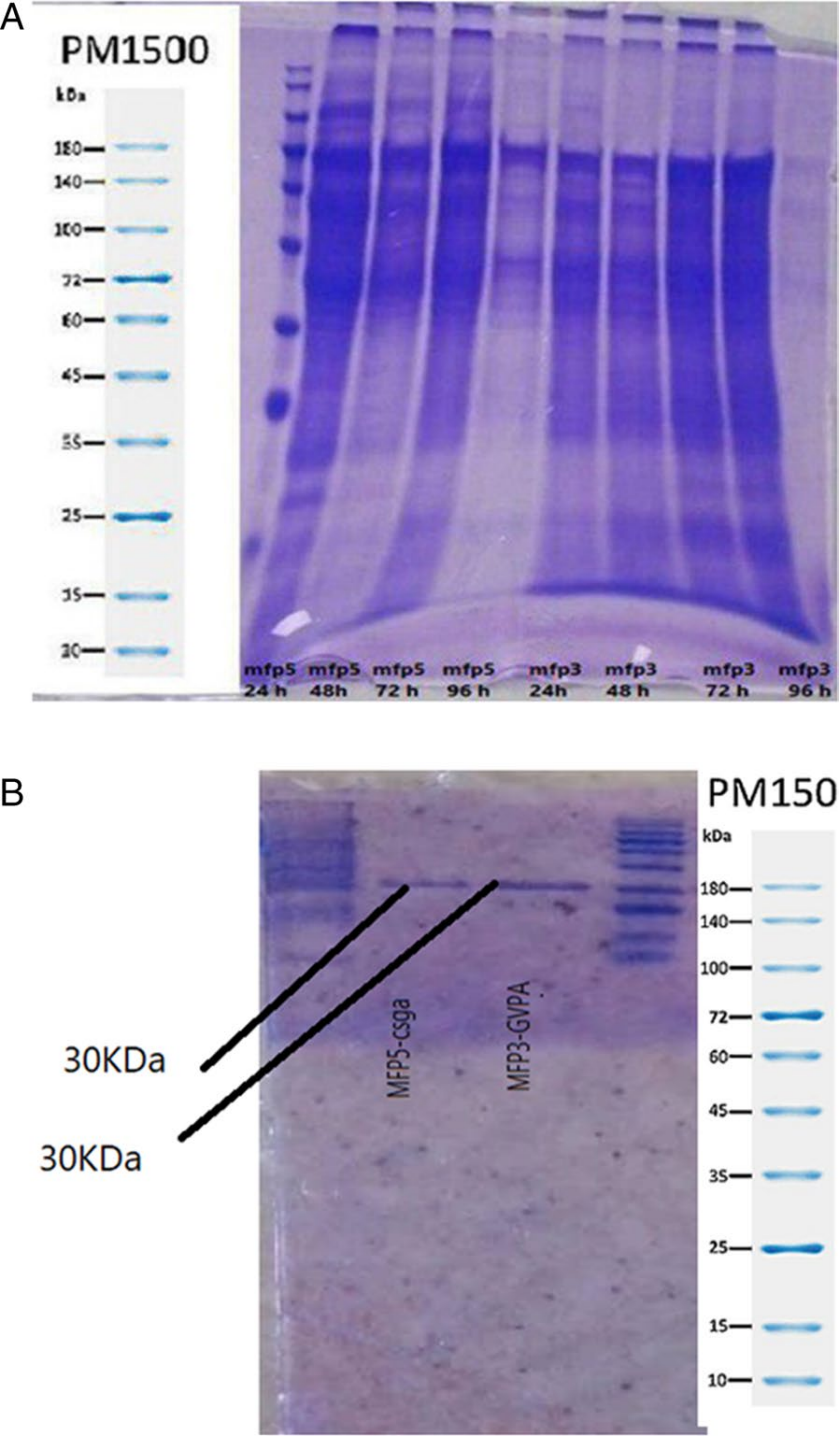
SDS-PAGE analysis for purified fused recombinant proteins before (**A**) and after purification (**B**)

We formerly expressed these chimeric proteins in prokaryotic *E. coli* expression system but here we exploited an endotoxin-free yeast host system for recombinant bio-adhesive protein production with more stable amyloid-like fibrils. Structurally similar from those produced in *E. coli*, recombinant proteins were expressed as inclusion bodies in yeast *Pichia pastoris.* After the induction of the recombinant proteins by adding 0.5% methanol each 24 h, cells were harvested, lysed and separated by SDS-PAGE 10% (Coomassie Brilliant Blue G-250 staining). HIS-Tag purification gave a purity of 90% for hybrid Mfp5-CsgA and Mfp3-GvpA (Fig. 2). Moreover, expression of bio-inspired amyloid bio-adhesive proteins after different times of incubation (24–96 h.) were compared in Fig. 2A. Data presented here revealed that current research matches the state-of-the-art chimeric proteins. Data presented here illustrated a rather higher expression in eukaryotic yeast system.

### Purification of recombinant proteins and western and analysis

**Fig. 3:**
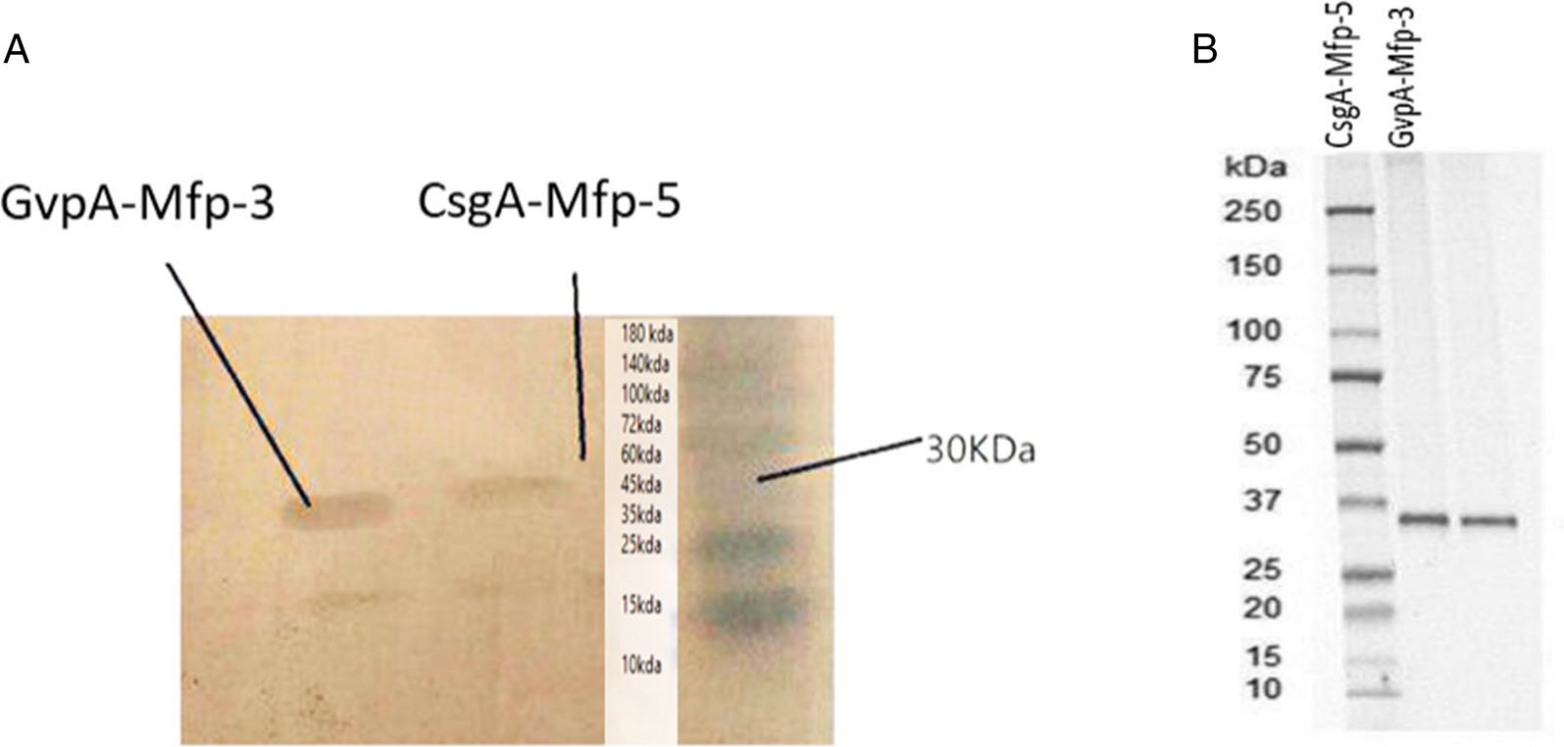
Western blotting analysis for recombinant chimeric proteins before and after purification (**A** and **B**)

Purification of recombinant proteins was done using nickel-containing affinity resin. Since chimeric proteins were not bound to the nickel resin due to the aggregation when they solubilized by 8 M urea, fresh 6 M guanidine HCl was used a stronger chaotropic agent as a protein denaturant for enhancing the solubility of recombinant proteins. After purification, Total purified proteins were analyzed with SDS-PAGE 10% (Coomassie Brilliant Blue G-250 staining) (Fig. 2C) and Western Blotting (Anti-histidine antibody) (Fig. 3). His-Tag purification gave a purity of 90% for hybrid Mfp5-CsgA and Mfp3-GvpA (Fig. 3). The presence of Mfp in each collected medium sample was confirmed by Western analysis. The Western blotting with specific polyclonal antibodies for each Mfp revealed two bands of approximately 32 and 31 kDa. Although all protein bands were clearly detected by Coomassie staining (Fig. 3), the bands of *P. pastoris*-derived Mfp5- CsgA and Mfp3-GvpA and modified *E. coli*-derived Mfp5 (Fig. 3) were observed on NBT-stained AU-PAGE except for unmodified *E. coli*-derived Mfp5-CsgA and Mfp3-GvpA. Thus, we were convinced that recombinant MAP from *Pichia pastoris* in vivo-modified DOPA.

### Mass spectrometry analysis

**Fig. 4:**
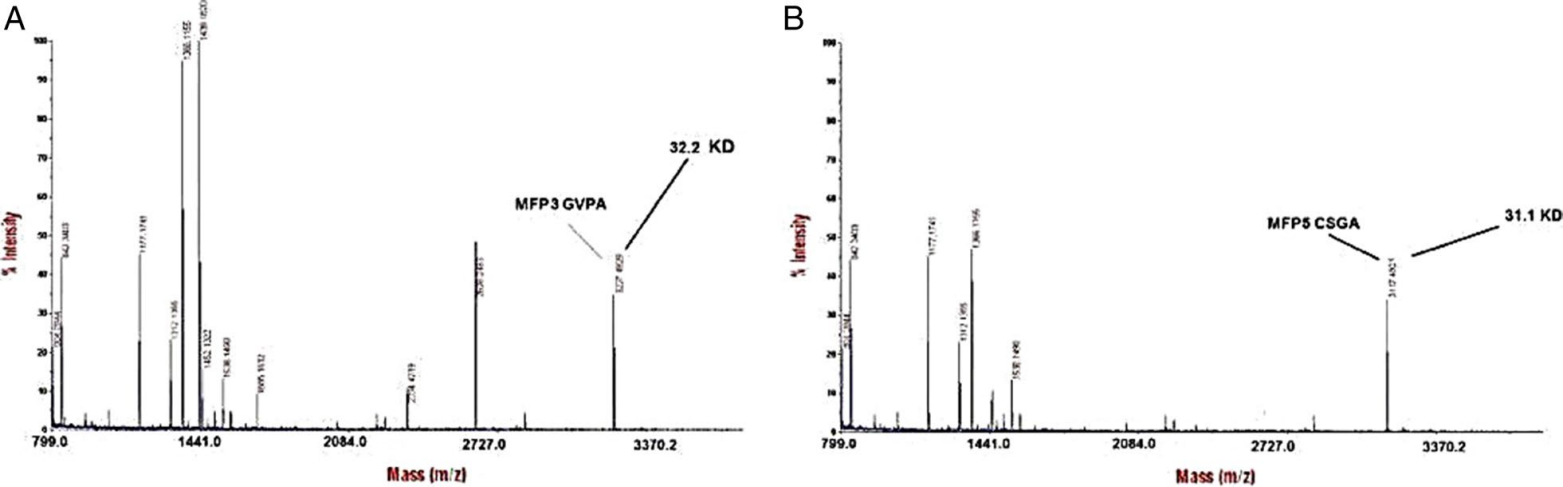
Purity and molecular weight of the adhesive proteins were determined by MS. MALDI-TOF analysis. Mfp3-GvpA (**A**), Mfp5-CsgA (**B**)

The MALDI-TOF analysis confirms the conclusion from SDS-PAGE that the Mfp3-GvpA is pure and intact. However, The MALDI-TOF spectrum for Mfp5 CsgA (Fig. 4) showed several peaks. The minor low mass peak at m/z 32,273 and 31,174 is in accordance with the expected molecular weight of the intact protein (31–32 kDa). This size of adhesive protein has been confirmed in other studies (Mori et al. 2007; Werten et al. 2019). The maximum concentration of the expressed Mfp5-CsgA and Mfp3-GvpA hybrid was 1.85 l g/mL and 1.92 g/mL in the 500-mL spinner flask culture, respectively.

### DOPA modification

**Fig. 5:**
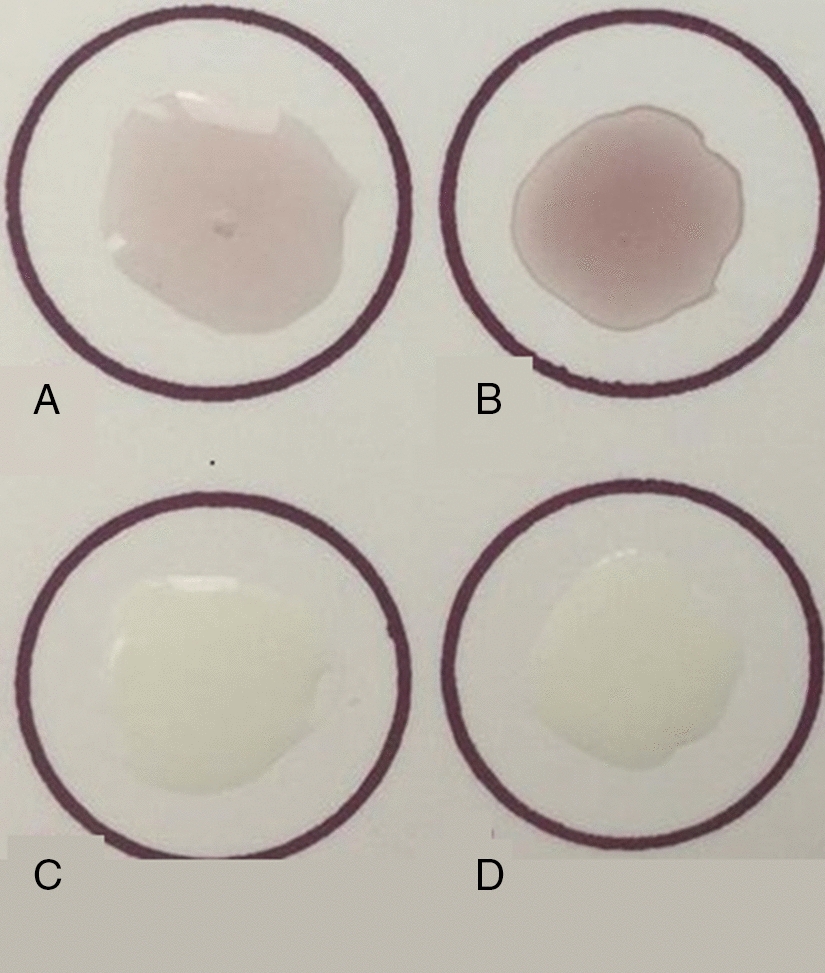
Redox-cycling nitro blue tetrazolium (NBT) assay for recombinant chimeric Mfp3-GvpA and Mfp3-GvpA adhesive proteins (100 μL of 40 μg/mL). Mfp3-GvpA after modification (**A**). Mfp5-CsgA after modification (**B**). Mfp3-GvpA before modification (**C**). Mfp5-CsgA before modification (**D**)

To confirm the presence of DOPA residues, acid-urea Polyacrylamide gel electrophoresis was performed with redox cycling staining involving nitro blue tetrazolium (NBT) and glycinate of each modified purified chimeric recombinant protein. For this purpose, first tyrosinase was added to the purified proteins followed by filtration by dialysis in 5% acetic acid and detection by NBT assay. As shown in Fig. 5A and B, all of the samples expressed in *P. pastoris* produced purple color demonstrating the conversion of tyrosine to DOPA. It is worth mentioning that the samples observed in Fig. 5C and D showed our unmodified samples of lack of expression in *Pichia pastoris* cells in which tyrosine had not converted to DOPA, resulting in the absence of purple color.

It is worth mentioning that the samples observed in figures C and D resulted from the expression of the above proteins in the bacterial host. Proteins that were expressed in bacteria were not completely modified; this test confirms that using eukaryotic yeast host cells to express adhesive proteins containing DOPA is better compared to that of expressed in prokaryotic hosts.

Further, the acid borate difference spectrum (ABDS) approach was performed to measure the quantity of conversion of Tyr to DOPA in purified chimeric recombinant proteins. DOPA quantity of Mfp5-CsgA and Mfp3-GvpA were obtained 73.2% ± 2.6 and 66.4% ± 3.5 respectively. Rather high levels of Tyr to DOPA conversion demonstrated strong adhesion property of the obtained recombinant proteins.

### Adhesion force measurement

**Fig. 6:**
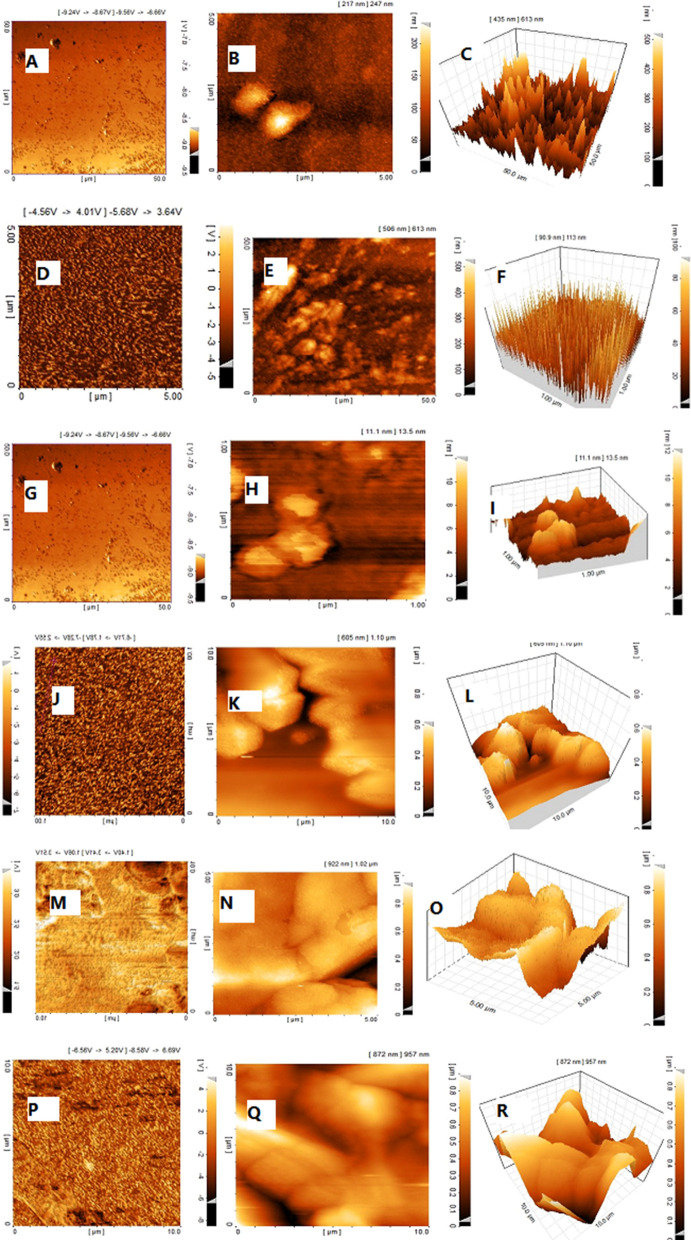
Surface morphology of Mfp3-GvpA and Mfp5-CsgA (**A**). Phase image of protein Mfp3- GvpA in PH 5.5 (**B**). topography image of Mfp3-GvpA in pH5.5 (**C**). 3D image of Mfp3-GvpA in pH 5.5 (**D**). Phase image of protein Mfp3-GvpA in pH 2.6 (**E**). topography image of Mfp3-GvpA in pH2.6 (**F**). 3D image of Mfp3-GvpA IN PH 2.6 (**G**). Phase image of protein Mfp5-CsgA in pH 5.5 (**H**). topography image of Mfp5-CsgA in pH5.5 (**I**). 3D image of Mfp 5-CsgA in pH 5.5 (**J**). Phase image of protein Mfp 5-CsgA in pH 2.6 (**K**). topography image of Mfp5-CsgA in pH2.6 (**L**). 3D image of Mfp 5-CsgA IN pH 2.6 (**M**). Phase image of protein Mfp3-GvpA and Mfp5-CsgA in pH 5.5 (**N**). topography image of Mfp3-GvpA and Mfp5-CsgA in pH5.5 (**O**). 3D image of Mfp3-GvpA, Mfp5-CsgA in pH 5.5 (**P**). Phase image of protein Mfp3-GvpA and Mfp5-CsgA in pH 2.6 (**Q**). topography image of Mfp3-GvpA and Mfp5-CsgA in pH 2.6 (**R**). 3D image of Mfp3-GvpA, Mfp5-CsgA in pH 2.6 (**S**)

In order to evaluate the adhesion force of each chimeric proteins and copolymer (in both unmodified and modified states), atomic force spectroscopy (AFS) was utilized with silica tip on mica surface in a wet condition. Due to the fact that the Mfp molecules existed in all specimens and both Mfp3 and Mfp5 have definite behaviors in different pH levels, the outcomes of transformations in phase, topography and 3D patterns are well categorized using atomic force microscopy (Fig. 6). In fact, the DOPA molecule in Mfp protein structure at pH 2.5 stimulates the extensive structure to better adhesion to the surface of the mica. Pertaining to the mentioned spectacles, by comparing the samples A to F with G to L and furthermore assessing the significant amounts of DOPA molecules in Mfp5 than the Mfp3, the variation between the pattern A and G can be explained.

Almost as the impact of GvpA hydrophobic monomers has a notable impact on the appearance of additional 3D peaks, this aspect serves more noticeably when relating C and F forms in lower pH scale. Compared to GvpA, CsgA, has two extra phenylalanine amino acids. CsgA is utilized to prepare DOPA and consequently further adherent to the surface, hence broadening the final structure. All of these, along with its fibrillar building, brings the 3D picture of the chimer molecule to appear more flats than GvpA-containing samples.

Finally, the mixture of all chimeric proteins offers a moderate structure and function in 2 and 3D angles and topography, which confirms the interactive behavior for each isolated chimeric protein. As shown in Fig. 6, the size of the Mfp5-CsgA protein is larger than Mfp3-GvpA and thus the latter has more potential to be used for biomedical applications though it requires more energy to separate from the mica surface. Besides the phase and topographic images of proteins Mfp5-CsgA and Mfp3-GvpA in the co-polymer state, Mfp5-CsgA has a larger structure compared to Mfp3-GvpA; this indicates that the adhesion force of Mfp5-CsgA is stronger than Mfp3-GvpA and the adhesion force in the polymer is far greater than that of Mfp3-GvpA protein. Also, the above proteins in acidic condition had more cohesion and density than in other pH conditions.

## Discussion

Considering the fact that most of the cells and tissues consist of 70% water which is negatively affecting adhesion performance of any bio-adhesive materials, various research teams have attempted to develop medical adhesives inspired by aquatic organisms adhesion. In spite of progresses in understanding the mechanistic details and crucial factors of the natural adhesion detected in aquatic organisms such as mussels, the production of feasible and functional bio-adhesives with strong wet adhesion ability is still one of the greatest challenges in the regenerative medicine industry (Lee et al. 2009; Jeon et al. 2015). This plays a key role in biofilm structure and adhesion to various surfaces. Various adhesive materials have been reported so far, but none of the products produced have been able to meet all the necessary requirements (Geurts et al. 2010; Hennebert et al. 2015). In the present study, the recombinant protein expressed in terms of the post-translational process was approved. Similar studies have also shown that *Pichia pastoris*, as a host, exhibits good expression of extracellular proteins mainly owing to post translation modifications and higher expression levels (Rueda et al. 2016; Zhu et al. 2018). The current study provides the first report of utilizing the yeast expression system to generate multiplex combinational fusion proteins composed of Mfp3, Mfp5, GvpA, and CsgA. Of course, previous studies reported the production of adhesive proteins in bacterial hosts, but the con of the bacterial system is the post-translational process (Waite 2017; Zhang et al. 2017a, b). An important advantage of using a fungal host is the post-translational process, such as proper glycosylation and secretion of the protein into the culture medium.

In prevailing literature, the role of DOPA as the main constituent for adhesion of mussel foot proteins has been reported (Mirshafian et al. 2016; Visekruna et al. 2015). In the present study, we exploited Mfp5 and Mfp3as prominent parts of the chimeric protein structure mainly due to their tyrosine residues present in the protein sequence which transform into DOPA for adhesion. Similar Studies have shown that these two proteins together can produce resistant bio-adhesives (Rubin et al. 2010) and proposed that they can be considered as favorable candidates for medical applications owing to their non-toxicity and weak immune response inductions (Zhang et al. 2017a, b). However, produced recombinant bio-glues are not mature enough and also have weak bonds, high cost, or low safety for clinical application (Kord Forooshani and Lee 2017). The production of novel recombinant proteins by combining them with curli adhesive fibers has been suggested as an efficient strategy for gaining better attachment and conjugation for better adhesion to reach the needs of the regenerative medicine market (Huang et al. 2012a, b).

We used the CsgA sequence from *E. coli* along with CsgA, another curli protein from *Cyanobacteria*, to improve adherence of Mfp3 and five other proteins. As the origin of CsgA sequence could be pathogenic, it may cause allergic sensitivities. Thus, we have assessed using another curli protein, GvpA, a non-pathogenic curli protein derived from cyanobacteria.

In order to improve the performance of our bio-adhesive in the present study, the curli sequence of proteins along with the main sequence of mussel foot adhesive proteins was used (Rahimnejad and Zhong et al*. *2017). obtained a new bio-adhesive protein by inserting the Mfp3-CsgA and Mfp5-CsgA genes through a single-step assembly into a bacterial cell. Mfp3-CsgA and Mfp5-CsgA were converted into fibril bundles due to their amyloid filaments, and the adhesive strength of this type of bi-adhesive in seawater was 20.9 mJ m-2, which was about 1.5 times higher than the ability of recombinant bi-adhesives produced at that time. The addition of cross-links to the adhesive structure can improve adhesion and its survival. Among the natural compounds that can be used as crosslinkers are the helixes of the proteins of early bacteria and *E. coli*. The recombinant protein Mfp5-CsgA has the ability to form a stable β structure in an aqueous solution so that Mfp will be present in the amyloid regions of CsgA in the central part (Waite and Qin 2001).

The two main practical challenges concerning adhesive protein expression, are low solubility of the purified protein and large-scale production (Wang et al. 2020; Stewart 2011). The usage of fungal hosts such as yeast, for the expression of various proteins, as opposed to prokaryotic systems has been reported to not cause an allergic response in human body (Huang et al. 2012a, b). To address the aforementioned issues, we used *P. pastoris* as a host for expression of the chimeric protein. The reason being its higher production rate and better post translational modifications necessary for efficient function of the obtained protein. Though there is definitely no ideal host for adhesive protein expression, our research shows that *Pichia pastoris* seems to be a quite suitable host due to the high glycosylation.

On the other hand, the structure and combinational features of chimeric proteins have a significant role in their characteristics (George and Carrington 2018). During the present study, the factors that have affected the efficiency and affinity of recombinant protein expression have been considered. Various culture conditions such as media component, temperature, pH, and incubation time have been modified to improve expression. The obtained data showed that expression of chimeric protein in lower temperature (25–26 °C) and with an additional 40% of YNB culture medium into the main culture medium (BMY) made for a more efficient approach for protein expression. This is compared to the prevailing approaches such as the ones stated in Invitrogen protocol (Safder et al. 2018). It might be due to the positive effect of low temperature and nutritional supplementation on protein expression and could be considered for large-scale heterogeneous protein performance (Silverman and Roberto 2017; Mori et al. 2007; Wang et al. 2020).

One of the major challenges in producing bio-adhesives from mussel foot proteinosis is the resistance demonstrated by adhesive proteins at a particular pH. In the present study, AFM microscopic images also show greater cohesion of proteins in acidic pH.

So far, many attempts have been made to produce bio-adhesives based on mussel foot proteins. However, it is unclear which protein is really the best choice for making bio-adhesives. Here, we assessed combination of Mfp3 and curli GvpA proteins as well as Mfp5 along with other curli CsgA proteins. AFM microscopic images revealed that the best strength and coherence of each of these proteins solely are obtained in acidic condition mainly owing to their high DOPA content. However, fusion of each Mfp protein with a curli protein led to improvement of the coherence and stability of the final structure. Altogether, it can be concluded that obtained adhesion strength of chimeric proteins Mfp3 and Mfp5 with curli protein CsgA and GvpA was significantly higher than that of native Mfp proteins. This is majorly due to the improved cross linking in recombinant Mfp3 and Mfp5 proteins by curli proteins. Moreover, post-translational modification of obtained protein in eukaryotic expression systems such as *P. pastoris* might have a positive effect on its adhesion capacity. Thus, we conclude that *P. pastoris* yeast would be considered as a suitable expression system for the expression of adhesive proteins such as Mfp3, Mfp5, CsgA and GvpA for large scale industrial bio-glue production. These bio-adhesive proteins capable of functioning in wet condition can be used in various fields of medicine including dentistry, surgery and drug delivery approaches.

## Data Availability

Not applicable.

## References

[CR3] Baumann K, Dato L, Graf AB, Frascotti G, Dragosits M, Porro D, Mattanovich D, Ferrer P, Branduardi P (2011). The impact of oxygen on the transcriptome of recombinant *S. cerevisiae* and *P. pastoris*—a comparative analysis. BMC Genomics.

[CR50] Cha HJ, Hwang DS, Lim S, White JD, Matos-Perez CA, Wilker JJ (2009). Bulk adhesive strength of recombinant hybrid mussel adhesive protein. Biofouling.

[CR4] Choi YS, Kang DG, Lim S, Yang YJ, Kim CS, Cha HJ (2011). Recombinant mussel adhesive protein fp-5 (MAP fp-5) as a bulk bioadhesive and surface coating material. Biofouling.

[CR5] Deming TJ (1999). *Mussel**byssus* and biomolecular materials. Curr Opin Chem Biol.

[CR6] Dolgin E (2013). The sticking point. Nat Med.

[CR7] George MN, Carrington E (2018). Environmental post-processing increases the adhesion strength of *Mussel**byssus* adhesive. Biofouling.

[CR8] Geurts P, Zhao L, Hsia Y, Gnesa E, Tang S, Jeffery F, La Mattina C, Franz A, Larkin L, Vierra C (2010). Synthetic spider silk fibers spun from Pyriform Spidroin 2, a glue silk protein discovered in orb-weaving spider attachment discs. Biomacromol.

[CR9] Hayes PK, Buchholz B, Walsby AE (1992). Gas vesicles are strengthened by the outer-surface protein, GvpC. Arch Microbiol.

[CR10] Hennebert E, Maldonado B, Demeuldre M, Richter K, Rischka K, Flammang P, Hennebert E, Maldonado B (2015). From sand tube to test tube: the adhesive secretion of Sabellariid tubeworms. Bio-adhesion and biomimetics from nature to applications.

[CR11] Huang Y, Zhang Y, Wu Y, Wang J, Liu X, Dai L, Mo W (2012). Expression, purification, and mass spectrometric analysis of 15N, 13C-labeled RGD-hirudin, expressed in *Pichia**pastoris*, for NMR studies. PLoS ONE.

[CR12] Huang Y, Zhang Y, Wu Y, Wang J, Liu X, Dai L, Wang L, Yu M, Mo W (2012). Expression, purification, and mass spectrometric analysis of 15N,13C-labeled RGD-hirudin, expressed in *Pichia pastoris*, for NMR studies. PLoS ONE.

[CR13] Iranpour H, Hosseini SN, Far HH, Zhand S, Ghanbarlu MM, Shahsavarani H, Bouzari S, Shokrgozar MA (2021). Self-assembling of chimeric mussel-inspired bio-adhesives originated from Mytilus californianus and Anabaena flos-aquae: a new approach to develop underwater adhesion. Int J Adhes Adhes.

[CR14] Jeon EY, Hwang BH, Yang YJ, Kim BJ, Choi BH, Jung GY, Cha HJ (2015). Rapidly light-activated surgical protein glue inspired by mussel adhesion and insect structural crosslinking. Biomaterials.

[CR15] Kord Forooshani P, Lee BP (2017). Recent approaches in designing bioadhesive materials inspired by mussel adhesive protein. J Polym Sci Part A Polym Chem.

[CR16] Lee H, Rho J, Messersmith PB (2009). Facile conjugation of biomolecules onto surfaces via mussel adhesive protein inspired coatings. Adv Mater.

[CR17] Mattanovich D, Graf A, Stadlmann J, Dragosits M, Redl A, Maurer M, Kleinheinz M, Sauer M, Altmann F, Gasser B (2009). Genome, secretum and glucose transport highlight unique features of the protein production host *Pichia**pastoris*. Microb Cell Fact.

[CR18] Mirshafian R, Wei W, Israelachvili JN, Waite JH (2016). α, β-dehydro-dopa: a hidden participant in mussel adhesion. Biochemistry.

[CR19] Mori Y, Urushida Y, Nakano M, Uchiyama S, Kamino K (2007). Calcite-specific coupling protein in barnacle underwater cement. FEBS J.

[CR20] Pfeifer F (2012). Distribution, formation and regulation of gas vesicles. Nat Rev Microbiol.

[CR21] Priemel T, Degtyar E, Dean M, Harrington M (2017). Rapid self-assembly of complex biomolecular architectures during *Mussel**byssus* biofabrication. Nat Commun.

[CR22] Rahimnejad M, Zhong W (2017). Mussel-inspired hydrogel tissue adhesives for wound closure. RSC Adv.

[CR23] Rebnegger C, Graf AB, Valli M, Steiger MG, Gasser B, Maurer M, Mattanovich D (2014). In Pichia pastoris, growth rate regulates protein synthesis and secretion, mating and stress response. Biotechnol J.

[CR24] Rubin DJ, Miserez A, Waite JH (2010). Diverse strategies of protein sclerotization in marine invertebrates: structure–property relationships in natural biomaterials. Adv Insect Physiol.

[CR25] Rueda F, Gasser B, Sánchez-Chardi A, Roldán M, Villegas S, Puxbaum V, Ferrer-Miralles N, Unzueta U, Vázquez E, Garcia-Fruitós E, Mattanovich D, Villaverde A (2016). Functional inclusion bodies produced in the yeast *Pichia pastoris*. Microb Cell Fact.

[CR26] Rzepecki LM, Hansen KM, Waite JH (1992). Characterization of a cystine-rich polyphenolic protein family from the blue Mussel *Mytilus**edulis* L.. Biol Bull.

[CR27] Safder I, Khan S, Islam IU, Ali MK, Bibi Z, Waqas M (2018). Pichia pastoris expression system: a potential candidate to express protein in industrial and biopharmaceutical domains. Biomed Lett.

[CR28] Silverman HG, Roberto FF (2007). Understanding marine mussel adhesion. Mar Biotechnol.

[CR29] Stewart RJ (2011). Protein-based underwater adhesives and the prospects for their biotechnological production. Appl Microbiol Biotechnol.

[CR30] Urushida Y, Nakano M, Matsuda S, Inoue N, Kanai S, Kitamura N, Kamino K (2007). Identification and functional characterization of a novel barnacle cement protein. FEBS J.

[CR31] Urushida Y, Nakano M, Matsuda S, Inoue N, Kanai S, Kitamura N, Nishino T, Kamino K (2007). Identification and functional characterization of a novel barnacle cement protein. FEBS J.

[CR32] Visekruna A, Linnerz T, Martinic V, Vachharajani N, Hartmann S, Harb H, Joeris T, Pfefferle PI, Hofer MJ, Steinhoff U (2015). Transcription factor c-Rel plays a crucial role in driving anti-CD40-mediated innate colitis. Mucosal Immunol.

[CR34] Waite JH (2017). Mussel adhesion—essential footwork. J Exp Bot.

[CR35] Waite JH, Qin X (2001). Polyphosphoprotein from the adhesive pads of *Mytilus edulis*. Biochemistry.

[CR36] Wang Q, Zhong C, Xiao H (2020). Genetic engineering of filamentous fungi for efficient protein expression and secretion. Front Bioeng Biotechnol.

[CR37] Werten M, Eggink G, Cohen Stuart MA, de Wolf FA (2019). Production of protein-based polymers in *Pichia**pastoris*. Biotechnol Adv.

[CR38] Yu J, Wei W, Danner E, Israelachvili JN, Waite JH (2011). Effects of interfacial redox in mussel adhesive protein films on mica. Adv Mater.

[CR39] Yu J, Wei W, Menyo MS, Masic A, Waite JH, Israelachvili JN (2013). Adhesion of mussel foot protein-3 to TiO2 surfaces: the effect of pH. Biomacromol.

[CR40] Zhang X, Huang Q, Deng F, Huang H, Wan Q, Liu M, Wei Y (2017). Mussel-inspired fabrication of functional materials and their environmental applications: progress and prospects. Appl Mater Today.

[CR41] Zhang W, Yang H, Liu F, Chen T, Hu G, Guo D, Hou Q, Wu X, Su Y, Wang J (2017). Molecular interactions between DOPA and surfaces with different functional groups: a chemical force microscopy study. RSC Adv.

[CR42] Zhao H, Waite JH (2006). Linking adhesive and structural proteins in the attachment plaque of *Mytilus**californianus*. J Biol Chem.

[CR43] Zhu W, Chuah YJ, Wang DA (2018). Bioadhesives for internal medical applications: a review. Acta Biomater.

